# GenAlEx 6.5: genetic analysis in Excel. Population genetic software for teaching and research—an update

**DOI:** 10.1093/bioinformatics/bts460

**Published:** 2012-07-20

**Authors:** Rod Peakall, Peter E. Smouse

**Affiliations:** ^1^Evolution, Ecology and Genetics, Research School of Biology, The Australian National University, Canberra ACT 0200, Australia and ^2^Department of Ecology, Evolution and Natural Resources, School of Environmental and Biological Sciences, Rutgers University, New Brunswick, NJ 08901-8551, USA

## Abstract

**Summary:** GenAlEx: Genetic Analysis in Excel is a cross-platform package for population genetic analyses that runs within Microsoft Excel. GenAlEx offers analysis of diploid codominant, haploid and binary genetic loci and DNA sequences. Both frequency-based (*F*-statistics, heterozygosity, HWE, population assignment, relatedness) and distance-based (AMOVA, PCoA, Mantel tests, multivariate spatial autocorrelation) analyses are provided. New features include calculation of new estimators of population structure: *G*′_ST_, *G*′′_ST_, Jost’s *D*_est_ and *F*′_ST_ through AMOVA, Shannon Information analysis, linkage disequilibrium analysis for biallelic data and novel heterogeneity tests for spatial autocorrelation analysis. Export to more than 30 other data formats is provided. Teaching tutorials and expanded step-by-step output options are included. The comprehensive guide has been fully revised.

**Availability and implementation:** GenAlEx is written in VBA and provided as a Microsoft Excel Add-in (compatible with Excel 2003, 2007, 2010 on PC; Excel 2004, 2011 on Macintosh). GenAlEx, and supporting documentation and tutorials are freely available at: http://biology.anu.edu.au/GenAlEx.

**Contact:** rod.peakall@anu.edu.au

## 1 INTRODUCTION

GenAlEx 6 was originally developed as a teaching tool to facilitate teaching population genetic analysis at the graduate level (Peakall and Smouse, 2006). GenAlEx operates within Microsoft Excel—the widely used spreadsheet software that forms part of the cross-platform Microsoft Office suite. Packaging genetic analysis within a familiar and flexible environment resulted in quick understanding and effective performance of population genetic analyses. Taking advantage of the rich graphical options available within Excel, GenAlEx offers a wide range of graphical outputs that aid genetic data analysis and interpretation. GenAlEx is now widely used by university teachers at both undergraduate and graduate levels around the world. Moreover, the software has also attracted a large number of researchers who utilize its unique features. Here we provide an update on the new features offered in GenAlEx 6.5 that we believe will be welcomed by students, teachers and researchers.

GenAlEx offers population genetic analysis of diploid codominant, haploid, haplotypic and binary genetic data from animals, plants and microorganisms. It accommodates a wide range of genetic markers, including microsatellites (SSRs), single-nucleotide polymorphisms (SNPs), amplified fragment length polymorphisms and DNA sequences. Both allele frequency-based and distance-based analysis options are provided. The former includes estimates of heterozygosity and genetic diversity, *F*-statistics, Nei’s genetic distance, population assignment and relatedness. The latter includes Analysis of Molecular Variance (AMOVA), Principal Coordinates Analysis (PCoA), Mantel tests, TwoGener, multivariate and 2D spatial autocorrelation. Readers are referred to Peakall and Smouse (2006) for a more comprehensive outline of these standard procedures, data formats and data import options.

GenAlEx 6.5 maintains backward compatibility, but it provides access to the expanded spreadsheet of Excel 2007 onward. Thus, the maximum numbers of loci and samples are vastly expanded and only constrained by memory. More than 30 different Excel graphs summarize the outcomes of genetic analyses. Graphics can be further manipulated with Excel options and easily converted to pdf or other publication-quality formats.

## 2 NEW FEATURES

### 2.1 New estimators of population structure

There has been much recent debate about the utility of *F*_ST_ as a measure of population genetic structure (Jost, 2008; Ryman and Leimar, 2009; Whitlock, 2011). GenAlEx 6.5 offers the calculation of *G′*_ST_, *G′′*_ST_ and Jost’s *D*_est_, providing [0,1]-standardized allele frequency-based estimators of population genetic structure, following Meirmans and Hedrick (2011), testing the null by random permutation and estimating variances via jackknifing and bootstrapping over loci. New AMOVA routines now enable the estimation of standardized *F′*_ST_, following Meirmans (2006). The calculation of these statistics was validated by comparison with the software GenoDive v2.0b22 (Meirmans and Van Tienderen, 2004).

### 2.2 Shannon’s information statistics

Shannon information indices have been widely used in ecology but largely overlooked in genetics despite offering a framework for quantifying biological diversity across multiple scales (genes to landscapes). GenAlEx offers the calculation of a series of Shannon indices, including the mutual information index *^S^H*_UA_, an alternative estimator of population structure. The methods follow Sherwin *et al.* (2006) who assessed the performance of Shannon indices for estimating genetic diversity. Smouse and Ward (1978) extend to multiple hierarchical levels, with a unique three-level partition option and statistical testing by random permutation offered in GenAlEx 6.5.

### 2.3 Tools for comparing pairwise population statistics

The Mantel test capability of GenAlEx has been extended to allow multiple comparison among pairwise population statistics such as *F*_ST_, *F′*_ST_, *G′*_ST_, *G′′*_ST_, *D*_est_ and *^S^H_UA_*. This will allow informed comparison of the new estimators of population structure.

### 2.4 Heterogeneity testing for spatial autocorrelation

GenAlEx 6.5 introduces novel heterogeneity tests (Smouse *et al.*, 2008), extending application of the multiallelic, multilocus spatial autocorrelation analysis methods of Smouse and Peakall (1999), Peakall *et al.* (2003) and Double *et al.* (2005). These new methods provide valuable insights into fine-scale genetic processes across a wide range of animals and plants. Banks and Peakall (2012) have confirmed the statistical power and performance of this heterogeneity test by spatially explicit computer simulations.

### 2.5 Linkage disequilibrium tests (LD) for biallelic data

Despite its importance, there is no universal test for disequilibrium (Slatkin, 2008). GenAlEx 6.5 offers pairwise tests for disequilibrium between biallelic markers such as SNPs. When phase is known, this includes the calculation of *D, D′*, *r* and *r*^2^, following Hedrick (2005). Maximum likelihood estimation is used to calculate *D* and *r* when phase is unknown (Weir, 1990, p. 310). The results were validated against GDA (Lewis and Zaykin, 2001). Inclusion of LD fills an important technical gap, particularly for teachers. For large SNP sets, or multiallelic data, GenAlEx users are encouraged to take advantage of the options to export their data to other packages such as Arlequin 3.5 (Excoffier and Lischer, 2010).

### 2.6 New allele frequency format

Retrospective calculation of the new estimators of population structure such as *G′*_ST_, *D*_est_ and Shannon indices are now possible from published allele frequency data. Teachers will also find this a helpful option for the re-analysis of textbook examples.

### 2.7 Import and export options

GenAlEx offers data import from several popular formats and tools for importing and manipulating raw data from DNA sequencers. Export to more than 30 other data formats is provided, enabling access to myriad other software packages. For example, direct export is offered to programs such as GENEPOP (Rousset, 2008) and STRUCTURE (Pritchard *et al.*, 2000), and via these same formats to many other programs, including genetic packages in R such as adegenet (Jombart, 2008) and pegas (Paradis, 2010). The full list of export options, along with notes on the export process, can found at the website.

## 3 SPECIAL FEATURES FOR TEACHING

Offering a user-friendly software package for university students and teachers remains an ongoing goal of GenAlEx. We continue to expand the popular step-by-step output options that allow students to follow the steps in the analytical pathway. Teaching-specific menu options are also provided. For example, the Rand menu allows students to permute and bootstrap hypothetical datasets with color tracking, to aid an understanding of how these statistical tests work. Finally, we have made freely available a set of tutorial notes and supporting datasets drawn from the graduate workshops that we have offered (both jointly and independently) around the world.

## 4 DOCUMENTATION

More than 150 pages of documentation are provided. This includes Appendix 1 that outlines the statistical analyses used and their supporting references. The revised guide to GenAlEx 6.5 fully cross-links with the GenAlEx tutorials and Appendix 1.

## 5 CONCLUSION

GenAlEx 6.5 offers a wide range of population genetic analysis options for the full spectrum of genetic markers within the Microsoft Excel environment on both PC and Macintosh computers. When combined with its user-friendly interface, rich graphical outputs for data exploration and publication, tools for data manipulation and export options to many other software packages, we believe that GenAlEx offers an ideal launching pad for population genetic analysis by students, teachers and researchers alike.
